# Structure and Stabilities of Solution and Gas Phase Protein Complexes

**DOI:** 10.1021/jasms.4c00306

**Published:** 2024-11-21

**Authors:** Robert L. Rider, Carter Lantz, Liqi Fan, David H. Russell

**Affiliations:** Department of Chemistry Texas A&M University College Station, Texas 77843, United States

## Abstract

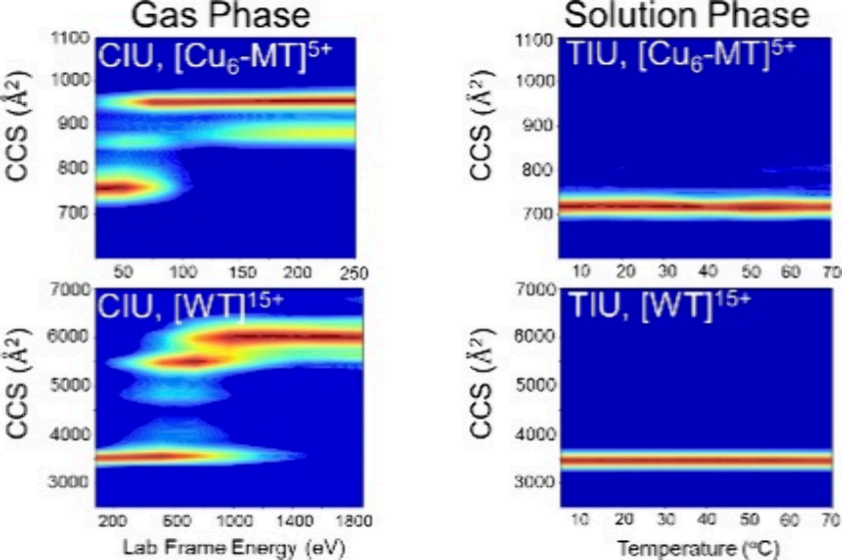

Collision-induced unfolding (CIU) has provided new levels of understanding of the stabilities and structure(s) for gas phase protein and protein complex ions formed by electrospray ionization (ESI). Variable-temperature (vT-ESI) data provide complementary information about temperature-induced folding/unfolding (TIU) reactions of solution phase ions. Results obtained by using CIU and TIU provide complementary information about stabilities of gas phase versus solution phase ions. Such comparisons may provide the most direct experimental approach to answer a long-standing question from Fred McLafferty: “For how long, under what conditions, and to what extent, can solution structure be retained without solvent?” Answers to this question require greater understanding of the (i) structure(s), stabilities, and dynamics of proteins/protein complexes in solution prior to ESI; (ii) effects of water removal by droplet fission and “freeze-drying” by evaporation of water from the nanodroplet; and (iii) potential reactions and structural changes that may occur as the ions traverse the heated capillary, the final stage in the transition to solvent-free gas phase ions. Here, we employ vT-ESI coupled with ion mobility-mass spectrometry (IM-MS) as a means to provide more detailed answers to the above-mentioned questions. Apo- and metalated-metallothionein-2A (MT), a cysteine-rich metal binding protein, and various proteoforms of transthyretin (TTR), a homotetrameric (56 kDa) retinol and thyroxine transporter protein complex were studied to examine distinct features of CIU and TIU across two different types of protein complexes. The results in this work shed light on the capabilities of CIU, TIU, and average charge state (Z_avg_) for probing the rugged energy landscape of native proteins and highlights the effects of water and cofactors (metal ions) on the structure and stabilities of proteins and protein complexes.

## Introduction

**Figure 1:**
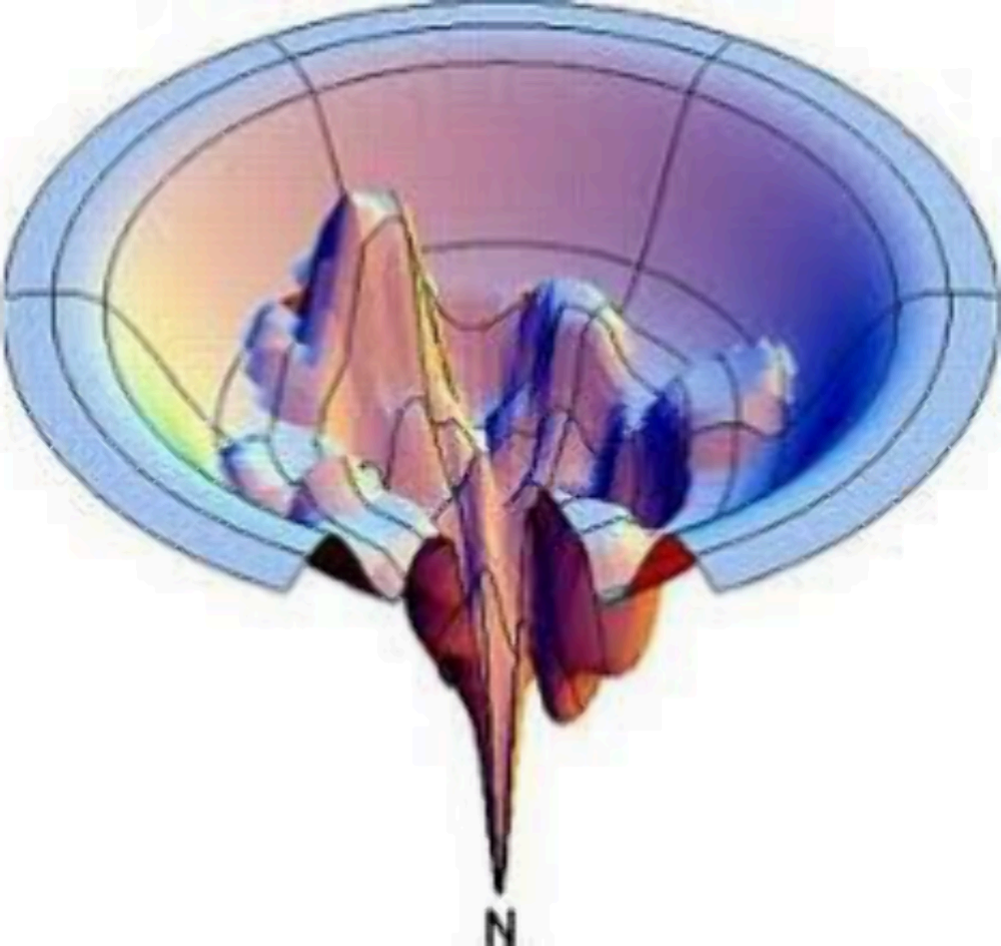
A rugged energy landscape for protein folding illustrating conformational entropy and deep energy wells that serve as kinetic traps. The well “N” defines the native state, in which many microstates exist, resembling the total rugged energy landscape. Reproduced with permission from ref (7). Copyright 1997 Springer Nature.

Structures, stabilities, and dynamics of proteins and protein complexes are influenced by interactions with other molecules, and water is an especially important participant in these folding and restructuring processes.^1^ Moreover, many of the effects on protein folding and restructuring reactions that are attributed to cofactor, osmolytes, and ligand binding are in fact direct effects of changes in hydration of the protein.^2^ Collectively, the effects of noncovalent interactions and hydration modulate the protein folding landscape by altering the contributions of enthalpy (Δ*H*) and entropy (*T*Δ*S*) to the energy folding landscape. Hydration stabilizes proteins by forming hydrogen bonds with polar amino acids on the protein surface, and temperature has direct influence on the dynamics of water that can promote restructuring processes, especially under cold and hot conditions.^3,4^ The combined effects of hydration and temperature on protein folding and restructuring are noteworthy, with the hydration of biomolecules being largely entropically driven.^5,6^ In the case of large, polar biomolecules, limited here to proteins and protein complexes, multiple low energy conformational states are possible, which is best illustrated by the “rugged energy landscape” (REL) (see Figure 1).^7,8^ The REL harbors rich conformational entropy (conformation states) in which kinetic or thermodynamic traps are present for “non-native” states (microstates)^9^ that may be “hidden” from experimental approaches that report ensemble averaged data, viz., isothermal titration calorimetry, X-ray crystallography, and cryo-electron spectroscopy.^10,11^ Understanding the roles and mapping the conformational entropy of the REL are important when considering that these native-like and non-native protein states may underly the aberrant behavior that may lead to malfunction and even disease.

There is a growing realization that electrospray ionization combined with ion mobility-mass spectrometry (ESI-IM-MS) has unique potential to provide more complete mapping of the protein REL,^12−15^ viz. effects of solution temperature, pH, ionic strength, presence/absence of cofactors/osmolytes and solvent on protein structure, stabilities, and dynamics.^13,16^ When combined with nano-ESI (nESI), which provides rapid cooling (130–150 K) of protein-containing nanodroplets described as “freeze-dried biomolecules”,^17^ IM-MS allows analysis of the kinetically trapped conformers present that make up the REL.^18^ Combining nESI with IM-MS affords measurements of mass-to-charge ratios, average charge state (Z_avg_), and size-to-charge ratios (collision cross section, CCS) of the ions. Moreover, variable-temperature with IM-MS (vT-ESI-IM-MS) otherwise known as temperature induced unfolding (TIU), can determine the effects of solution temperature within the ESI emitter (from ∼2–98 °C) on Z_avg_, ion abundances, and changes in CCS of reactants and products.^19^ Continued development of techniques such as TIU, may allow for greater insight into solution phase dynamics of biomolecules such as the ones in this work.

Native mass spectrometry (nMS) studies comparing solution and gas phase ions have a rich history;^20−23^ however, the importance of the stabilization effects of water in solution and the transition of ions from solution to the gas phase is less understood.^14,20,24−26^ Collision-induced unfolding (CIU) is used to compare stabilities of gas phase ions (intact proteins and protein complexes) by measuring changes of the CCS as the collisional activation energies are increased.^20−23^ CIU of gas phase ions is primarily an enthalpically driven process, whereas vT-ESI (cold and hot) reports on conformational changes that are induced by changes in temperature and hydration, both of which can be from enthalpy and entropy driven processes.^1,3,4,27−30^ As a general rule, cold temperatures promote the formation of compact ions, whereas higher temperatures favor extended conformers. An exception is the phenomenon of cold unfolding, in which the increased hydration of hydrophobic residues promotes formation of extended conformers.^3,31,32^ CIU of the gas phase ions can be directly monitored with IM-MS by monitoring changes in the CCS of the ions. Conformational changes induced by changes in solution temperature can also be determined by IM-MS, but oftentimes changes in CCS are too small to be detected; see REL (Figure 1) and note that the activation energies for transitioning among the available states lie well below that for escaping the population of native-like states.^7^ Temperature-induced conformational changes may result in changes of the solvent accessible surface area (SASA) which can be detected by changes in Z_avg_, thereby revealing the presence of microstates (viz. REL) within the native and native-like states._._^19,24,25,33−36^ The results of this study further illustrate the complementary information obtained using CIU and TIU, and may yield new strategies for better understanding the linkage between structures of solution and gas phase ions.

## Experimental Section

### Materials

Equine cytochrome c, equine myoglobin, bovine ubiquitin, ammonium acetate, LC-MS grade water, LC-MS grade methanol, tris(2-carboxyethyl) phosphine hydrochloride (TCEP), pentetic acid (DTPA), cadmium acetate, copper acetate, and silver acetate were purchased from Millipore-Sigma (Burlington, MA). LC-MS grade acetic acid was purchased from Thermo Fisher (Waltham, MA). Bio-Spin 6 kDa MWCO SEC desalting columns were purchased from Bio-Rad Laboratories (Hercules, CA) and used for buffer exchanges.

### Protein Expression and Preparation

Human metallothionein-2A (MT) was expressed and purified as previously described.^37^ Aliquots of apo-MT (15 μM) were buffer exchanged into a 50 mM ammonium acetate solution with 1 mM TCEP and 10% %v/v methanol. The metalation of MT has been previously discussed, but briefly Cd_4_, Ag_4_, and Cu_6_-MT were prepared by titration of apo-MT with metal-acetate solutions.^38^ Tetrameric wild-type transthyretin (wt-TTR) was expressed and purified as previously described by Shirzadeh et al.^39^ Multiple TTR mutants were expressed and purified using the same method starting from different constructs. All TTR samples were buffer exchanged into 200 mM ammonium acetate and diluted to 5 μM for charge state measurement and 10 μM for CCS measurement to ensure a better signal. For TTR vT-ESI on the Thermo Fisher Q-Exactive UHMR (Santa Clara, CA), TTR aliquots were diluted in half with 1 M ammonium acetate and 3 mM DTPA before the buffer was exchanged into 200 mM ammonium acetate to remove zinc ions.

### Ion-Mobility Mass Spectrometry

CIU experiments were performed as previously described for the Waters (Manchester, UK) traveling wave ion-mobility mass spectrometry (TWIMS) Synapt G2 instrument using nESI^38,40^ with instrument tuning conditions that minimize unwanted collisional activation of the initially formed ions (Table S1).^20^ The ions of interest were subjected to collisional activation (CA) prior to IM-MS via trap collision voltage. Different trap collision energies were applied by changing the voltage drop (increments of 5 V for MT and 15 V for TTR) between the exit of the quadrupole and the entrance to the TWIG region, which was filled with argon. The activation energies are reported as the laboratory frame collision energy, which was calculated as the product of the ion charge and trap collision voltage. CA data was compiled as CIU heat maps, using CIUSuite2 software^40,41^ creating two-dimensional plots of CCS versus laboratory frame energy using the 2D smoothing method with smoothing window size and iterations as 5 and 1, respectively. The *x*-axis (lab frame energy) was interpolated by using an axis scaling factor of 2. CIU50 values were extracted from the CA data by using the standard fitting routine. Standard feature detection parameters were 3 minimum feature length, 0 maximum CV gap length, and 50 and 100 feature allowed width for MT and TTR, respectively. Standard CIU50 parameters used were max spectral centroiding mode, 0 maximum CV gap length, and 50 and 200 transition region padding for MT and TTR, respectively. The CCS in nitrogen buffer gas were determined using a TWIMS calibration of cytochrome c, myoglobin, and ubiquitin in water/methanol/acetic acid (49:49:2) as described previously for each different instrument tuning.^42^

### Variable Temperature Mass Spectrometry

Variable-temperature experiments were performed using a 3-stage thermoelectric chip (Peltier) static nESI device that can modulate temperatures from (5–80 °C ± 2 °C) that has been described elsewhere.^19^ Temperatures were measured in sequential order from cold to hot in 5 °C increments, with the nESI emitter equilibrated at each temperature for 3 min prior to any measurement. All the vT- IM-MS (TIU) measurements were performed on a Waters Synapt G2 instrument using low gas phase activation tuning (Table S2). TIU heatmaps were generated using CIUSuite2 with the same software parameters stated above for plotting CIU heatmaps. Average charge states were measured using a ThermoFisher Q-Exactive UHMR instrument with parameters stated in supplemental Table S3. The TTR data was analyzed using the Z_avg_ function in Unidec,^43^ while MT data was analyzed manually using the relative abundances of the 4+ and 5+ charge states using the Z_avg_ equation discussed elsewhere.^44^

## Results and Discussion

Here, CIU and TIU are used to compare the structure(s), stabilities, and dynamics of gas phase and solution phase MT and TTR ions. The protein complexes were chosen for this study because they represent two different but important types of biological systems that have been extensively studied by many different research groups using very different experimental approaches.^38,39,45−53^ Our approach focuses on answering two questions related to the questions proposed by Fred McLafferty: (i) how are the physiochemical properties of these complexes influenced by solvent and (ii) are solution phase structures retained by the gas phase ions?^20,54^ The CIU studies on MT are designed to access the effects of specific cofactors (i.e., metal ions) on conformational stabilities that can then be compared to solution thermal stabilities. vT-ESI studies may answer questions such as does solution temperature-dependent changes (cold vs hot) alter the coordination of the metal ion? While numerous MT metalated products are formed by reactions with Cd^2+^, Ag^+^, and Cu^+^, we will focus on the cooperatively formed Cd_4_-MT^5+^, Ag_4_-MT^5+^, and Cu_*x*_-MT^5+^ (x = 4, 6, and 10).^38,40,47^ The TTR studies are limited to wt-TTR and V122I, L55P, V30M, F87A, and T119M mutants. The role of water on the structure and stabilities of wt- and mutant-TTR has been investigated by others,^45,53,55,56^ and it has been found that each of these mutants has very different stabilities under certain environmental conditions. Moreover, our studies focus on comparing stabilities in cold versus hot water and what happens when that water is removed.

### Stabilities of Metallothionein-2A

**Figure 2:**
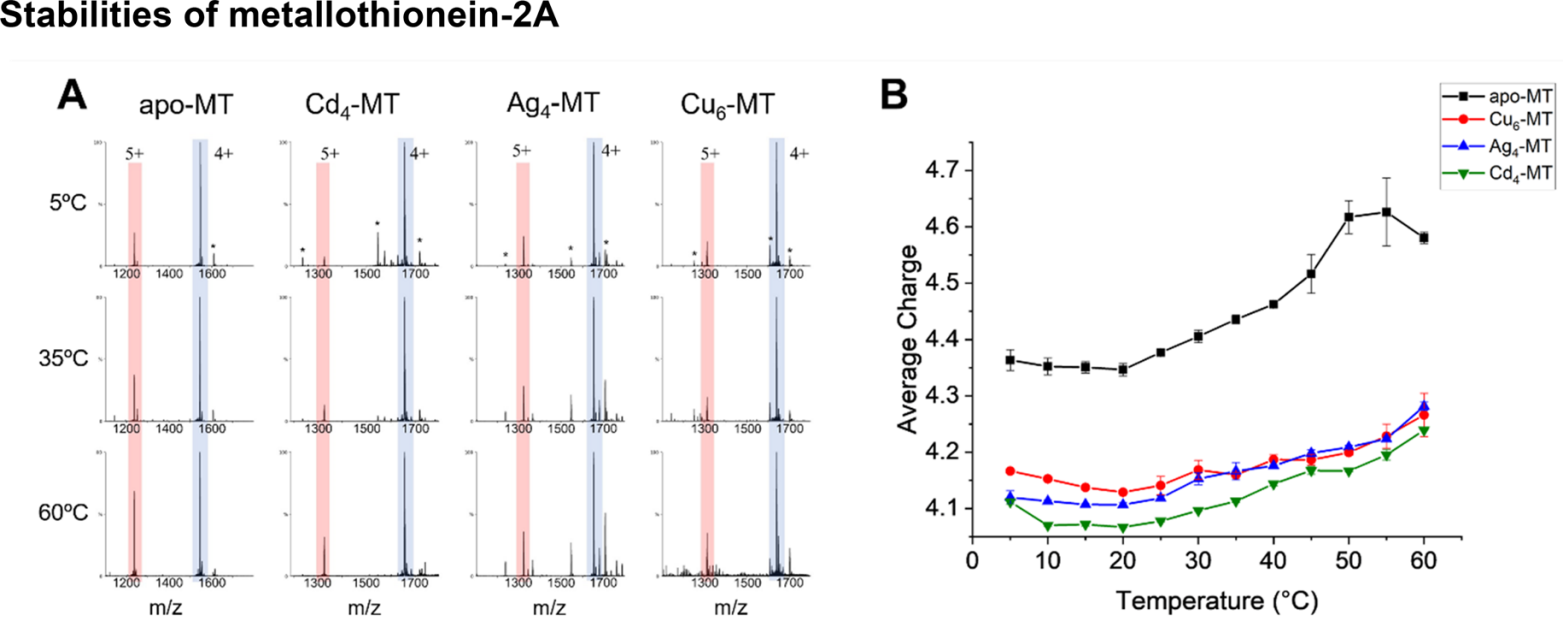
(A) Mass spectra of apo- and metalated MT at selected temperatures; 5^+^ ions are labeled in red and 4^+^ ions are labeled in blue. The asterisks (*) denote unreacted apo-MT and low-abundance metalated products for the MT^5+^ ions (Cd_7_, Ag_6_, Cu_4_, and Cu_10_). (B) Average charge states for apo- and partially metalated MT ions of the highest abundance species in the spectra; error bars represent standard deviations of replicates with the same nESI emitter (*n* = 3).

Figure 2A contains mass spectra for apo- and metalated (Cd^2+^, Ag^+^ and Cu^+^) MT-2A. The metalation of MT by Cd^2+^, Ag^+^ and Cu^+^ exhibits a high degree of cooperative binding as shown previously,^37,38^ and these spectra were taken at points in the metal titration where product ion abundances were at a maximum. Figure 2B contains Z_avg_ plots for apo- and metalated product ions as a function of nESI emitter solution temperature (5–70 °C). The effect of temperature on Z_avg_ for apo-MT is much larger compared to that of the metalated MTs (Cd_4_-MT Ag_4_-MT and Cu_6_-MT). Moreover, the increase in Z_avg_ with temperature provides evidence for changes in conformation or SASA even upon metalation. The decrease in Z_avg_ from 55–60 °C was only observed for apo-MT, which may be indicative of some significant structural rearrangement at these higher temperatures such as annealing. Deviation in Z_avg_ for apo- and metalated-MTs are indicative of differences in SASA and apo-MT having the highest Z_avg_ is likely due to the higher conformational entropy of apo-MT. The various metalated-MTs exhibited very similar solution thermal stability in the Z_avg_ experiment. These data provide evidence that metalation is most important to MT’s stability rather than the composition of metals bound.

**Figure 3:**
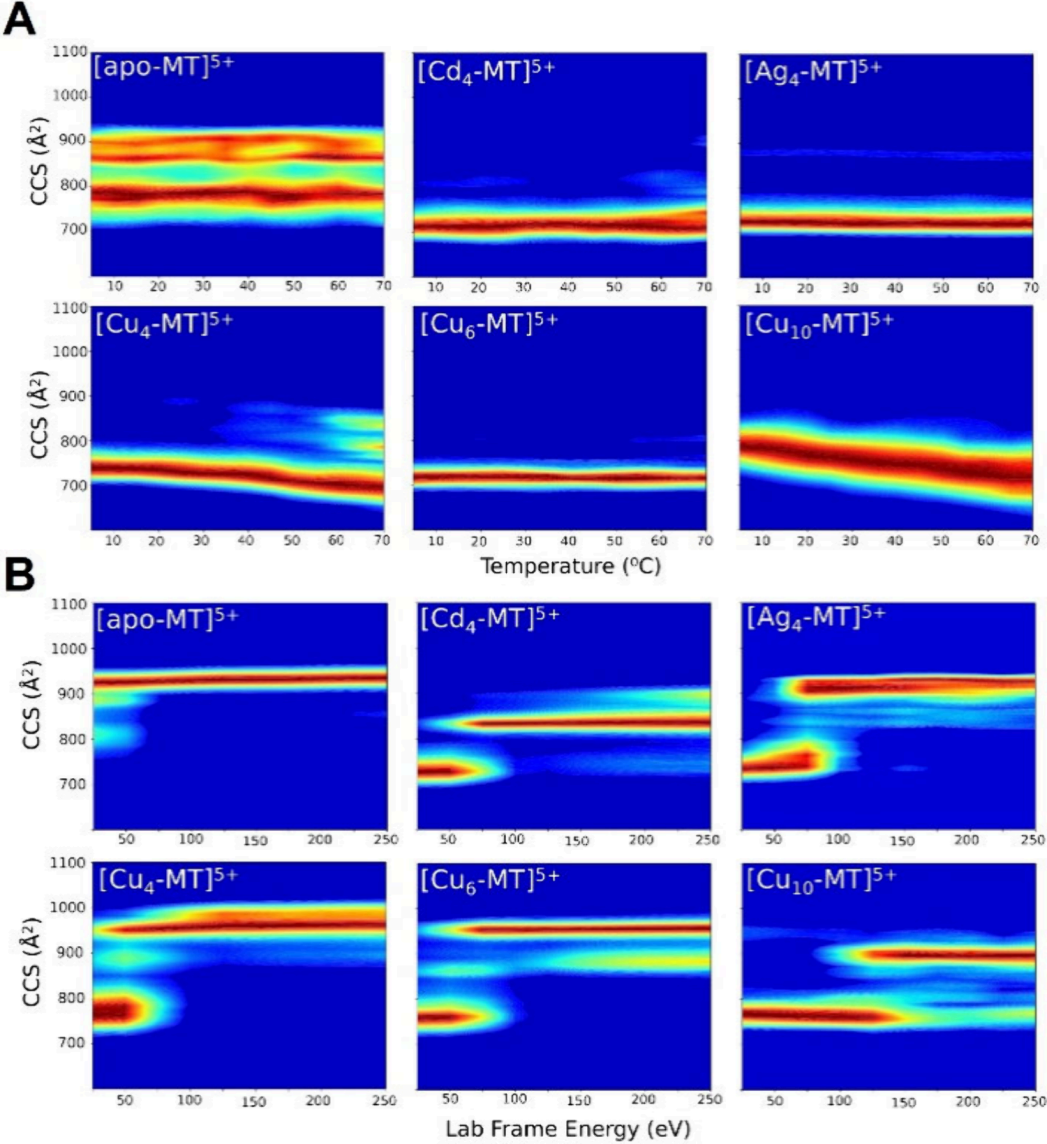
(A) TIU heat maps (5–70 °C) for 5^+^ apo-MT, Cd_4_-MT, Ag_4_-MT, Cu_4_-MT, Cu_6_-MT, and Cu_10_-MT. CCS profiles used to generate the TIU heat maps are shown in the Supporting Information (Figure S1). (B) CIU heat maps for apo- and metalated MT with raw 5+ CCS profiles used to generate data are shown in the Supporting Information (see Figure S2). For each of the metalated MTs CIU, there appears to be two main ion populations, one family of conformers with CCS centered at ∼750 Å^2^ and a larger CCS that differs for each metal. These differences are related to the cooperative metal coordination within the α- and β-domains.^37,38^

Figure 3 contains plots for CCS vs thermal activation and collisional activation for apo-MT^5+^ and metalated-MT^5+^ ions shown as CIU plots, and the CCS profiles used to generate these plots are shown in Figures S1 and S2. TIU data in Figure 3A show that apo-MT has two distinct conformations across the entire temperature range, which is not observed for any of the metalated-MT, underscoring the important role of metal binding on the conformational entropy for MT-metal product ions. This is consistent with the changes observed for Z_avg_ for all metalated MT discussed vide supra. The fwhm of the TIU CCS profiles for Cu_4_-, Cu_10_-, and Cd_4_-MT increase with temperature by approximately 7%, 30%, and 28%, respectively (Figure S1). These trends are very different for Cu_6_- and Ag_4_-MT as their CCS profiles decrease in fwhm (decreased conformational entropy), indicating that certain coordination numbers and metal types yield different solution thermal stabilities. Moreover, the Cu_4_-MT and Cu_10_MT complex is annealed or adopts a molten globule state that then forms more compact conformations, but at temperatures above ∼60 °C low abundance extended conformers are formed for Cu_4_-MT (see Figure 3A).^57^ This observation suggests that these metal bound states can adopt numerous Cu^+^ coordinated configurations. We previously showed that Cu_4_-MT is formed by addition of 4 Cu^+^ ions to the α-domain and Cu_10_-MT followed by addition of 6 Cu^+^ ions to the β-domain.^38^ Evidently, the dynamics of the metalated α- and β-domains are not coupled, and the peak broadening at high temperatures is evidence for restructuring of the complex at higher temperatures. Data for apo-MT^4+^ and metalated-MT^4+^ ions are shown in Figure S8; note that the thermal unfolding and collisional activation of 4^+^ ions have negligible effects on the CCS. The different TIU profiles for MT^4+^ and MT^5+^ are further evidence for solvent induced conformational differences. Reason(s) for these differences are unknown; consequently, the following discussion will be limited to the 5^+^ ions.

The CIU heat maps in Figure 3B indicate that MT gas phase stability increases upon metalation. Each metalation (number and metal type) differs in their final unfolded CCS, which is dependent on the metal coordination within the α- and β-domains.^38,40^ CIUSuite2 CIU50 values were extracted to compare initial to final conformer transition energies (see Figures S5 and S7A).^41^ Cu_10_-MT shows the highest CIU50 value at 137.6 eV, compared with apo-MT that is unfolded from the beginning (<25 eV) and all other metalated-MT had a CIU50 of ∼62 eV. These data show there is no significant reduction in unfolding with different metal coordination (Cd^2+^, Ag^+^, and Cu^+^), but the presence and stoichiometry of metal binding can increase MT’s gas phase stability.

The MT data provide evidence that gas phase and solution phase techniques sample different unfolding pathways along the REL for protein complexes. CIU data reveal that apo and metalated MT unfold readily with minimal collision energy. This indicates that the structure of the MT is unstable in the gas phase. Conversely, the TIU data reveal that the protein does not unfold significantly at any of the temperatures sampled; indirect evidence that water has stabilizing effects on the complex. It is important to note, however, that the conformational entropy does increase for certain MT-metal complexes (Cd_4_-, Cu_4_-, and Cu_10_-MT) with increased temperature observed with TIU and all MT-metal complexes for Z_avg_ measurements. This indicates that differences in interaction with water molecules alter the conformational entropy of the MT proteoforms. It is therefore possible that stabilization of the MT structure in solution is due to the surrounding water molecules that interact with the protein. The phenomenon of water molecule stabilization has been shown for many other protein–protein and protein–ligand systems.^2,55^ This suggests that entropic contributions by solvent play a part in stabilizing the MT structure, preventing the unfolding of MT and MT-metal complexes under destabilizing conditions (higher temperatures), and revealing the importance of water for the stabilization of protein-metal complexes that has not been studied in the current literature.

### Stabilities of WT and Mutant TTR

**Figure 4:**
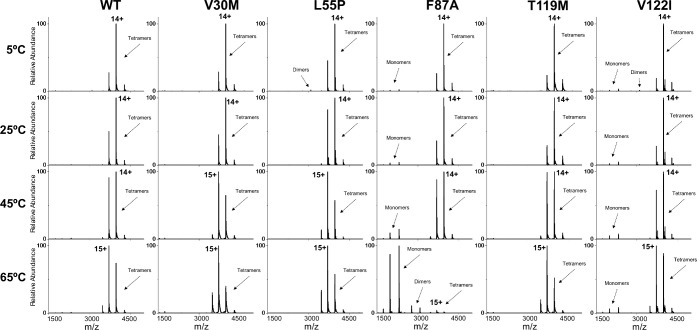
Mass spectra of wt- and mutant TTR at various temperatures using the vT-ESI device. Tetramers, monomers, dimers and the highest charge state of the tetramers are labeled in the spectra. F87A and V122I are the only mutants with significant amounts of monomer and/or dimer present at any temperature. Note that F87A consists primarily of monomers at 65 °C due to its low tetramer stability relative to the other TTR mutants.

The following experiments are designed to better understand the structure(s), stability, and dynamics of intact wt-TTR and TTR mutants. We and others have shown that under certain experimental conditions (pH, ionic strength, and temperature) wt-TTR and TTR mutants disassemble to form monomers and dimers.^39,51,53,55^ The results in this work provide evidence for the disassembly of TTR at various temperature values. At 80 °C, wt- and mutant TTR complexes disassemble to form abundant monomers (Figure S10), while the F87A mutant completely disassembles to monomers and dimers at temperatures of ∼65 °C (see Figure 4), and V122I has some low abundance of monomers throughout the temperature range examined.

**Figure 5:**
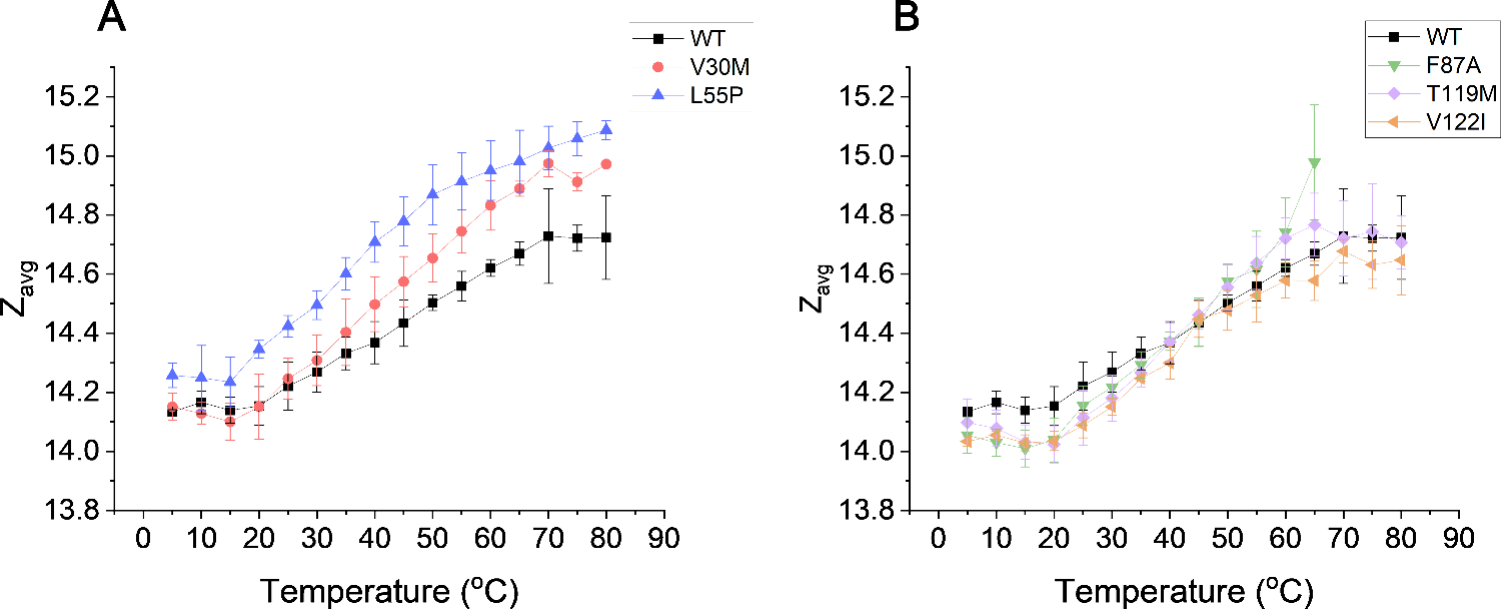
Average tetramer charge states of wt- and mutant TTR from 5 to 80 °C (or 65 °C for F87A because it completely dissocates at higher temperatures (Figure S10)). (A) Average charge states of wt-TTR and mutants located on the peripheral of TTR’s structure (Figure S9). Note that these mutants (V30M and L55P) have significantly higher charge states observed at higher temperatures. (B) Average charge states of wt-TTR and mutants located at interfaces (monomer and dimer) on TTR’s structure (Figure S9). Error bars represent the standard deviation of replicates with different nESI emmitters (*n* = 3). For both (A) and (B), wt- TTR data are the same.

**Figure 6:**
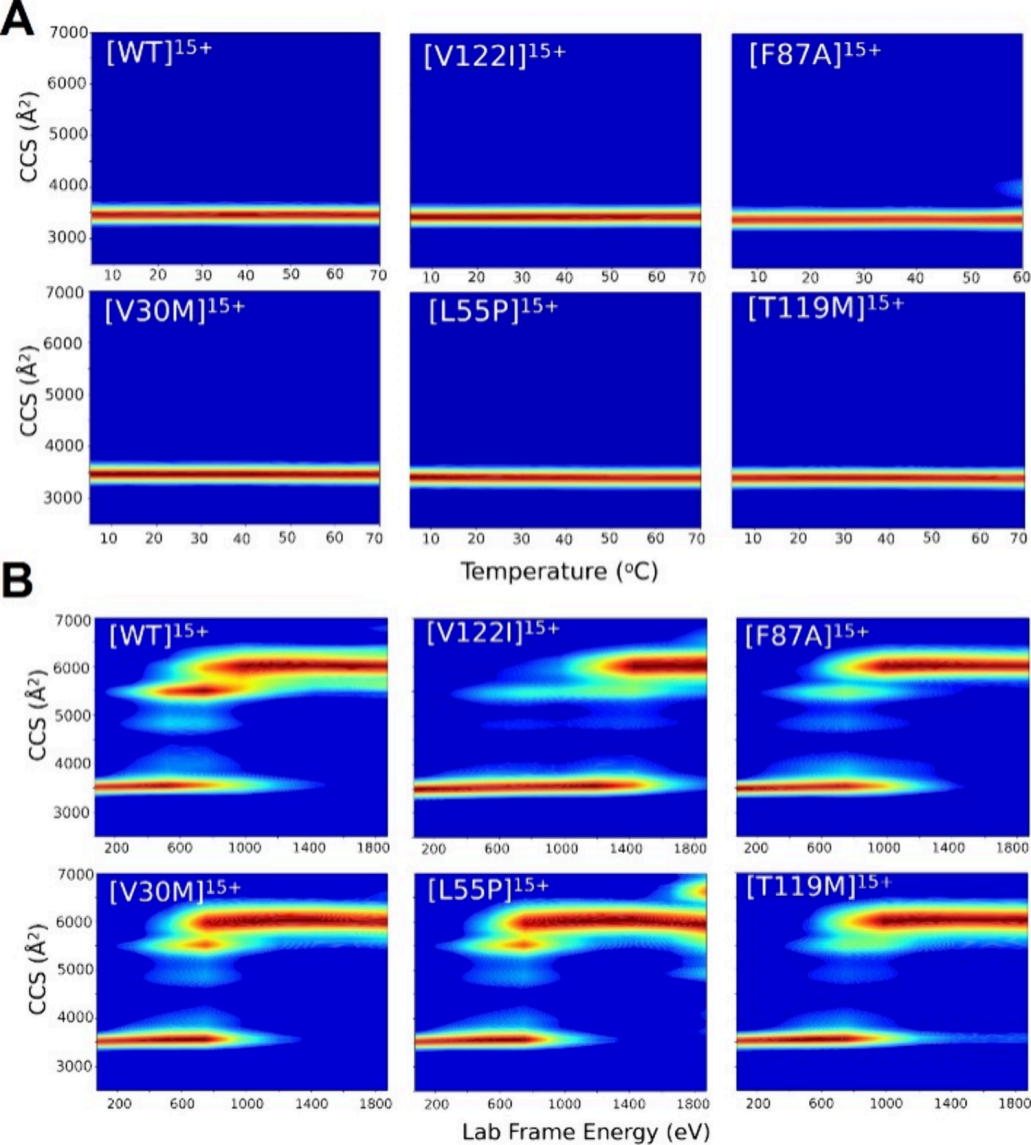
(A) TIU heatmaps of wt-TTR and TTR mutants (15^+^). There are no changes in CCS across the temperature range, demonstrating that TTR exhibits strong thermal stability in solution. (B) CIU heatmaps of wt-TTR and TTR mutants (15^+^). wt- and mutant TTR all unfold completely with the collisional activation provided; however, the energy required and intermediate pathways vary for each proteoform. Nuances in the CIU profiles are indicative of minor structural changes induced by point mutations.

Z_avg_ has been shown previously to examine nuanced changes in structure that may not be resolvable using IM-MS.^19^ The Z_avg_ values for WT-TTR and TTR mutants change significantly in the temperature range of 5 to 80 °C indicating there is a change in SASA when the solution temperature is changed (see Figures 5 and 6A). Notably, there is a slight increase in Z_avg_ at the colder temperatures for all samples (<20 °C), which we interpret as evidence of cold unfolding due to hydration of the nonpolar regions pertinent to protein folding.^3^ V30M and L55P exhibit a higher Z_avg_ relative to wt-TTR (Figure 5A), which may be related to their location on the peripheral part of the structure (see Figure S9). Changes in residue orientations around the TTR mutation sites have been observed with ^1^H NMR previously.^56^ Therefore, with V30M and L55P mutations being more exposed to the bulk solvent, the mutation-induced structural changes likely result in charge-carrying site (Z_avg_) differences. Additionally, L55P is more solvent exposed relative to V30M which could be why it exhibits the highest Z_avg_. Conversely, F87A, V122I, and T119M are all located at interfaces;^58^ thus, changes in residue orientations are not likely to result in changes in charge sites and the resulting Z_avg_ (see Figure 5B).^59^

CIU analysis provides information about the relative enthalpic stabilities of the TTR proteoforms. CIUSuite2 CIU50 analysis of the CIU data shows V122I requires the highest energy to unfold the native conformation (1367.7 eV) suggesting greater overall stabilities in the gas phase compared to V30M, L55P, F87A and T119M (see Supporting Information Figures S6 and S7B). Also, two typical intermediate states of CCS are observed in CIU experiments (denoted as I_1_ and I_2_) at ∼4000 and ∼4500 Å^2^ respectively (see Figure 6B), indicating that the unfolding involves reorganization of similar domains within the complex for all TTR proteoforms.^40,51^ Note that all TTR mutants have weaker intensity for these two intermediate states, respectively, with CIU compared to the wt-TTR, reflecting that these mutants have more unstable intermediate states and the conformational changes from the compact form to the fully unfolded form are more direct. Figure S4 shows the presence of these intermediate states arise early in the unfolding process of TTR mutants, but the relative abundances of the compact and fully unfolded states are always higher than for the intermediate states.

Although related in terms of physiological role/function, V122I, L55P, and V30M behave differently in CIU experiments. Figures 6B and S7B show that the compact form of V122I is retained at the highest collision energy and unfolds at a CIU50 of 1367.7 eV, but L55P and V30M dissociate at collision energies of 803.5 eV. L55P also shows additional unfolding at 1875 eV with the presence of a more extended conformer, indicating a difference in the unfolding pathway between wild-type and mutants (Figures 6B and S4). CIU profiles for T119M and F87A are most similar, with a CIU50 of 918.6 and 918.5 eV, respectively. The F87A mutant is known to be unstable in solution compared to the other mutants which may be the reason this proteoform unfolds at low energy; however, T119M is known to be more stable than the other mutants and wt-TTR- in solution. The instability of T119M in the gas phase suggests that water molecules are required to stabilize the structure of the mutant. Because the water molecules are removed in the gas phase, the stability of the T119M mutant decreases and it unfolds at low energy. This hypothesis is supported by a previous study that suggested the T119M mutation recruits more water molecules to a C-terminal loop region relative to wt-TTR.^53^ Furthermore, this observation may also indicate that the effect solvation has on structure and stability may be greatest for T119M compared with all the TTR species analyzed in this study. Here we show that CIU profiles can be utilized to probe the gas phase stability of TTR mutants and may provide information on solution phase characteristics of protein structure such as the role of hydration through comparison with TIU and Z_avg_.

The Z_avg_ shifts point to conformational and stability differences between wild type and mutant TTR; however, the extent of unfolding induced by collisional activation is much greater than the extent of unfolding observed by TIU. Wild type and mutant TTR all exhibit CCSs of ∼3500 Å^2^ across all temperatures (Figure 6A). A marginal amount of unfolding induced by TIU can be seen for the unstable mutant F87A at 60 °C; however, the tetramer dissociates past this temperature, and no mobility can be captured (see Figures 6A and S3). The thermal stabilities of the TTR tetramer complexes illustrate the importance of water for this system. Banerjee et al. reported that the hydrogen bond network of TTR is stabilized by eight water molecules that are surrounding the protein,^45^ and Ferrão-Gonzales et al.^60^ reported the importance of hydration and pressure in stabilizing for TTR; however, water molecules are not retained by the gas phase ions leading to understandable differences in solution and gas phase stabilities.

## Conclusion

Here, we compare gas phase and solution phase stabilities as well as conformational preferences for two protein complexes using CIU and TIU. CIU of gas phase MT and TTR proteoforms results in unfolding of protein ions, while TIU of the same proteoforms does not induce significant unfolding at temperatures between 5 and 80 °C. We attribute the observed thermal stability of the proteoforms analyzed in this study to known effects of hydration, as water molecules are known to play important roles in the stabilization of TTR^45^ and metalated proteins.^61^ Although unfolding is not apparent in TIU experiments, changes in Z_avg_ for MT and TTR are. We attribute this effect to slight rearrangement of protein complex structure due to alteration of the hydration surrounding the complexes in solution. These small changes in hydration induce changes in SASA but are too small to be detected by ion mobility mass spectrometry. The kinetic-trapping of such conformational shifts as a function of temperature (Figure 1) provides insight into the first question proposed in this study on whether the structure, stability, and dynamics of proteins can be monitored with nMS by providing evidence that nMS can sample microstates within the REL.^54^ The ability to unfold the protein, only after sufficient gas phase activation (CIU) provides evidence for Fred McLafferty’s second question that nMS is retaining native states as we transition from solution to gas phase.^54,62^ Future nMS studies should utilize CIU and TIU complementarily and decide which technique would provide the answer to their biological questions.

The results provide further rationale for utilizing native mass spectrometry for interrogating “native protein states” vis-à-vis “native-like protein states”.^7^ While the significance of how these states differ may be questioned, the role(s) for dynamic sampling of the protein folding landscape (conformational entropy) has important implications for structural biology. For example, water promotes hydrophobic interactions and interactions with metals,^63^ which serve to stabilize proteins, protein–ligand complexes, and their interactions with other biomolecules.^2^ Stabilizing water interactions may be why metal binding to MT decreases conformational entropy as seen in our TIU, CIU, and Z_avg_ data. Moreover, the Z_avg_ vs temperature plots for each of the three MT-metal binding states are indistinguishable, indicating that the different metal ions (Cd^2+^, Ag^+^, and Cu^+^) do not have significant influence on the solution phase conformational entropy relative to one another. Additionally, TTR is known to be stabilized by water, thus removal of hydrating water is expected to destabilize TTR, an effect that is observed when collisionally activating proteins in the gas phase with CIU. Collectively, these data further illustrate the stabilizing effects of water for protein complex structures and suggest that solvent effects must be accounted for when making inferences about protein structure.

## Supporting Information Available

Additional information for the TIU and CIU collision cross section profiles and CIUSuite2 analysis; instrument parameters for all experiments and samples; X-ray crystal structure with labeled mutant location for TTR (PDF)
