# Supplementary material for: Structure and Stabilities of Solution and Gas Phase Protein Complexes
Source: J Am Soc Mass Spectrom. 2024 Nov 21;35(12):3028–36. doi: 10.1021/jasms.4c00306 (PMC11622221; doi:10.1021/jasms.4c00306)
Supplement: Supplementary file 1 — js4c00306_si_001.pdf [file js4c00306_si_001.pdf]

Supporting Information

Structure and Stabilities of Solution and Gas Phase Protein Complexes

Robert L. Rider, Carter Lantz, Liqi Fan and David H. Russell*
Department of Chemistry
Texas A&M University
College Station, TX 77843

*Corresponding author: russell@chem.tamu.edu

Table of Contents

Figures	Page
S1. CCS profiles for apo- and metalated MT in TIU experiments.	S2
S2. CCS profiles for apo- and metalated MT in CIU experiments.	S3
S3. CCS profiles for wt- and mutant TTR in TIU experiments.	S4
S4. CCS profiles for wt- and mutant TTR in CIU experiments.	S5
S5. CIUSuite2 CIU50 plots for apo- and metalated MT in CIU experiments.	S6
S6. CIUSuite2 CIU50 plots for wt- and mutant TTR in CIU experiments.	S7
S7. CIUSuite2 CIU50 tabulated data for MT and TTR in CIU experiments.	S8
S8. CIUSuite2 heatmaps of CIU and TIU MT 4+ ions.	S9
S9. TTR X-ray crystallography structure denoting mutation locations.	S10
S10. TTR vT-ESI-MS on UHMR at 80 ^{o}C.	S11
Table S1. Synapt G2 parameters used for TIU experiments.	S12
Table S2. Synapt G2 parameters used for CIU experiments.	S13
Table S3. UHMR parameters used for vT-Z_{avg} experiments.	S14
References	S14

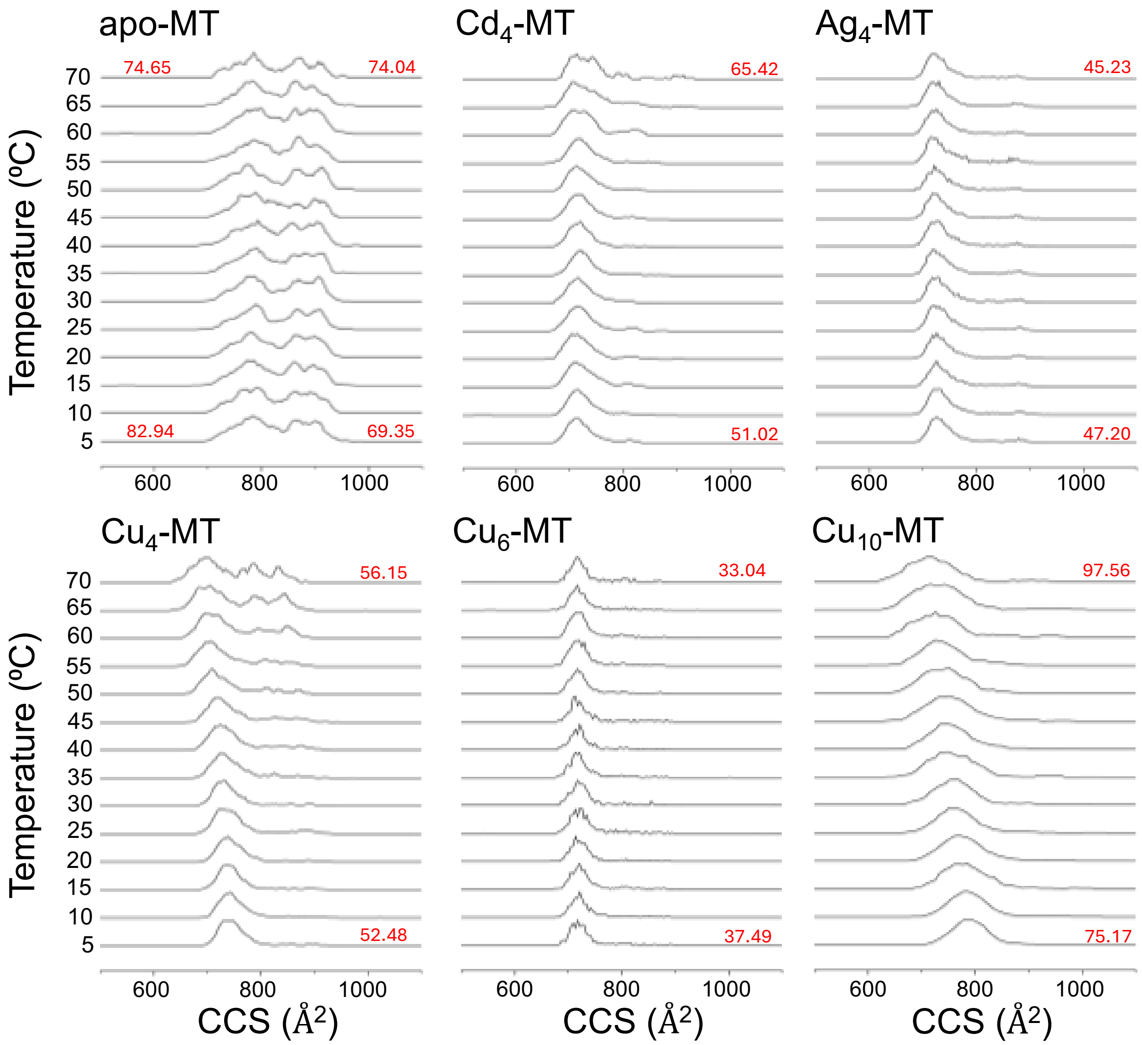

Figure S1. CCS profiles of 5+ apo- and metalated-MT from TIU experiments. The FWHM of the dominant conformer(s) are shown in red for 5 and 70 ºC. Apo-MT has two dominant CCS features and values are reported at both temperatures for the features. The FWHM was extracted using the peak deconvolution tool in OriginPro (OriginLabs, Northampton MA) using the 1st derivative method and searching for 1-2 peaks depending on the MT species. Percent change between conformer FWHM from 5 to 70 ºC is as follows: apo (-10.00%, 6.76%), Cd_4 (28.22%), Ag_4 (-4.17%), Cu_4 (6.99%), Cu_6 (-11.87%) and Cu_{10} (29.79%).

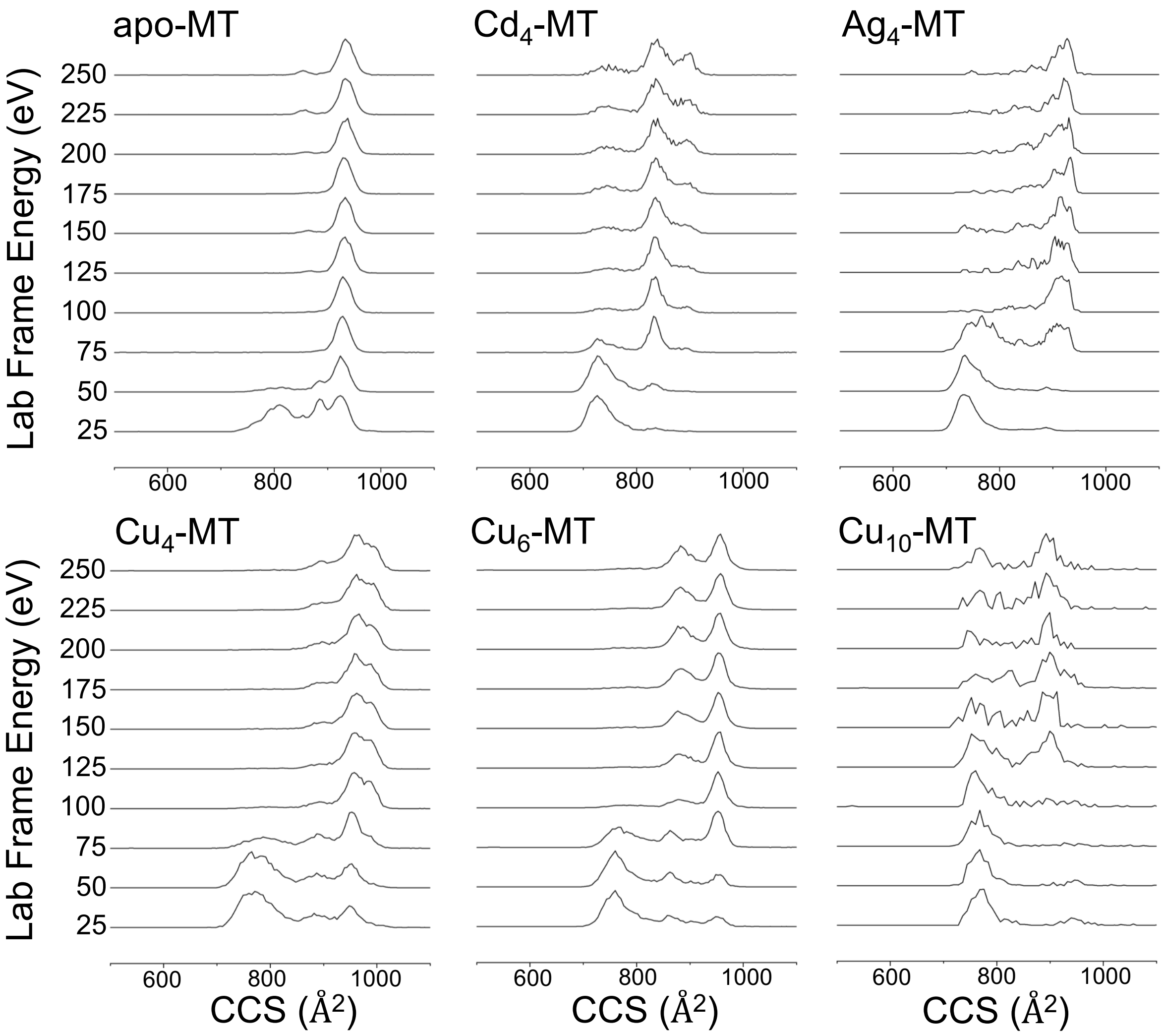

Figure S2. CCS profiles of 5+ apo- and metalated MT from CIU experiments.

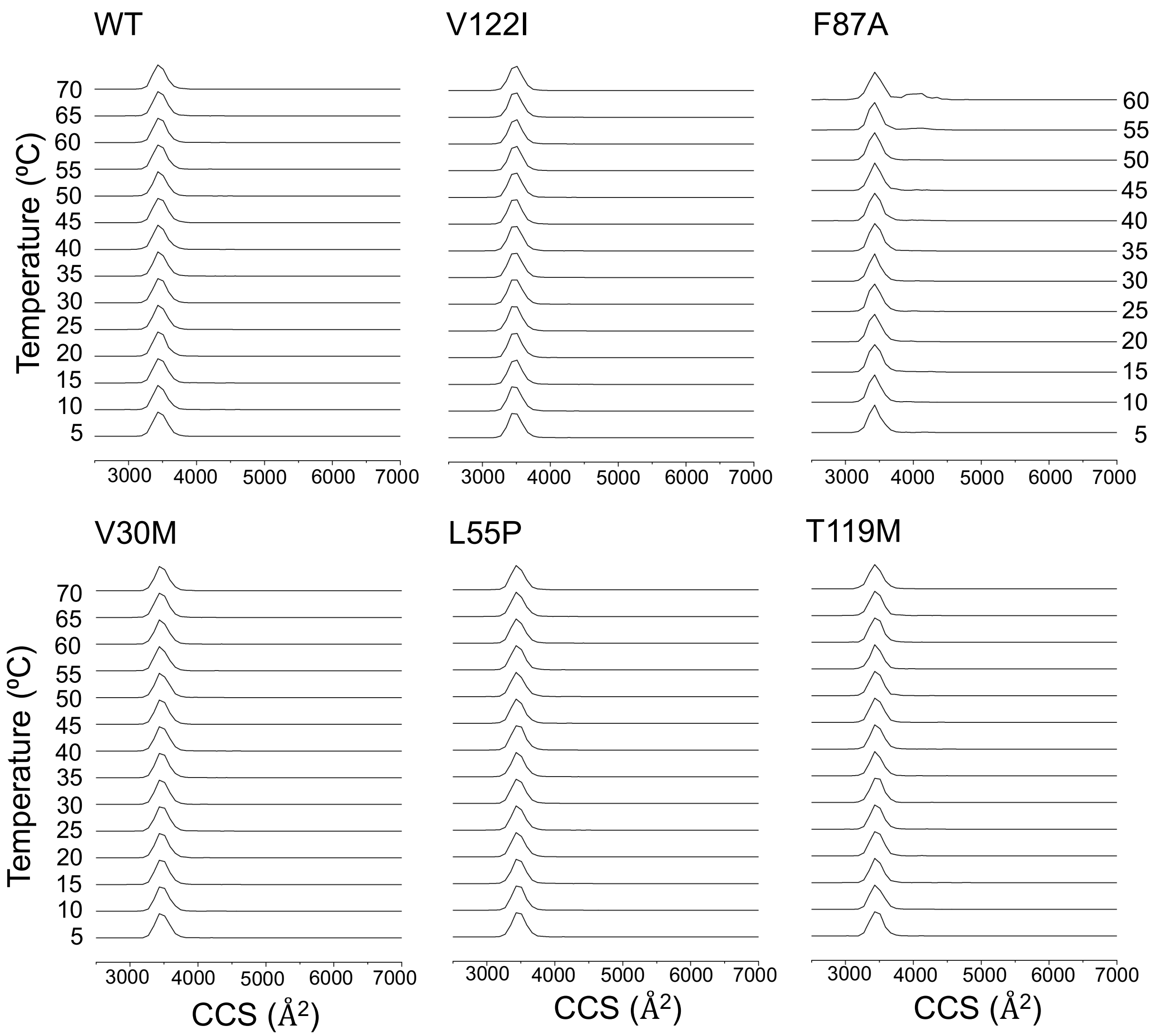

Figure S3. CCS plots of wt- and mutant TTR from TIU experiments. CCS plots are for the intact 15+ TTR tetramer ions, and do not include ions formed by disassembly of tetramer to smaller oligomers or monomers.

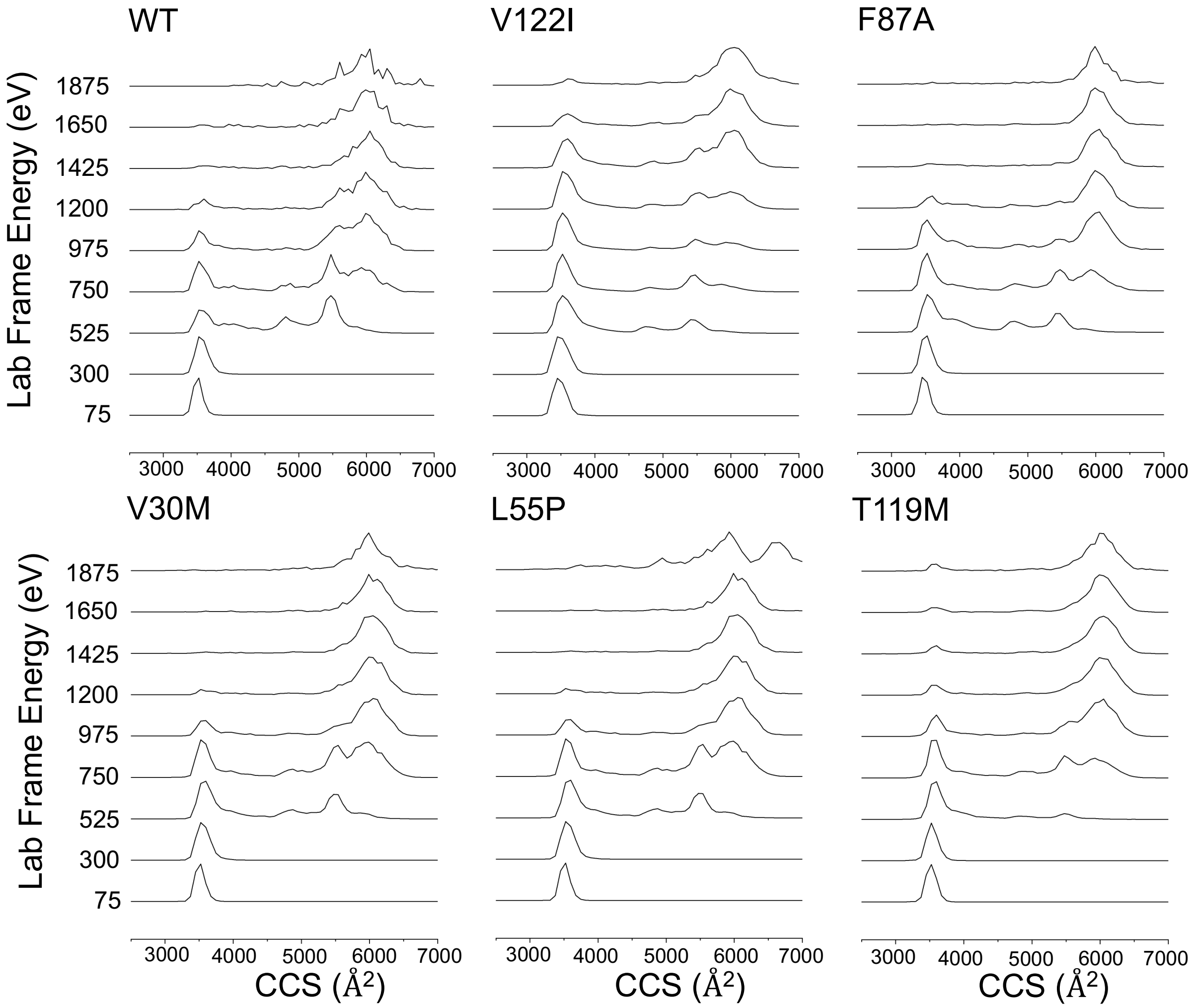

Figure S4. CCS plots of wt- and mutant TTR from CIU experiments. CCS plots are for the intact 15+ TTR tetramer ions, and do not include ions formed by disassembly of tetramer to smaller oligomers or monomers.

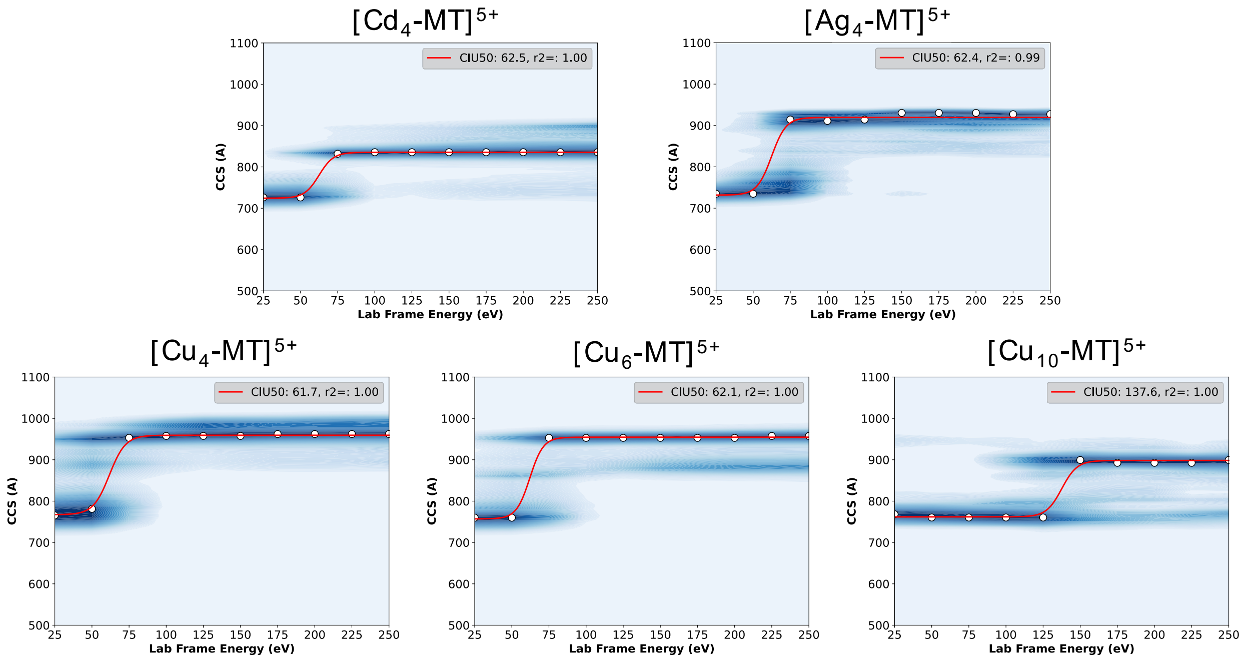

Figure S5. CIU50 plots of metalated-MT 5+ ions from CIU experiments using the standard fitting method in CIUSuite2. There was no reasonable fit for the apo-MT data due to the low abundance across few energy frames of the lower CCS feature, therefore not allowing a CIU50 to be calculated.

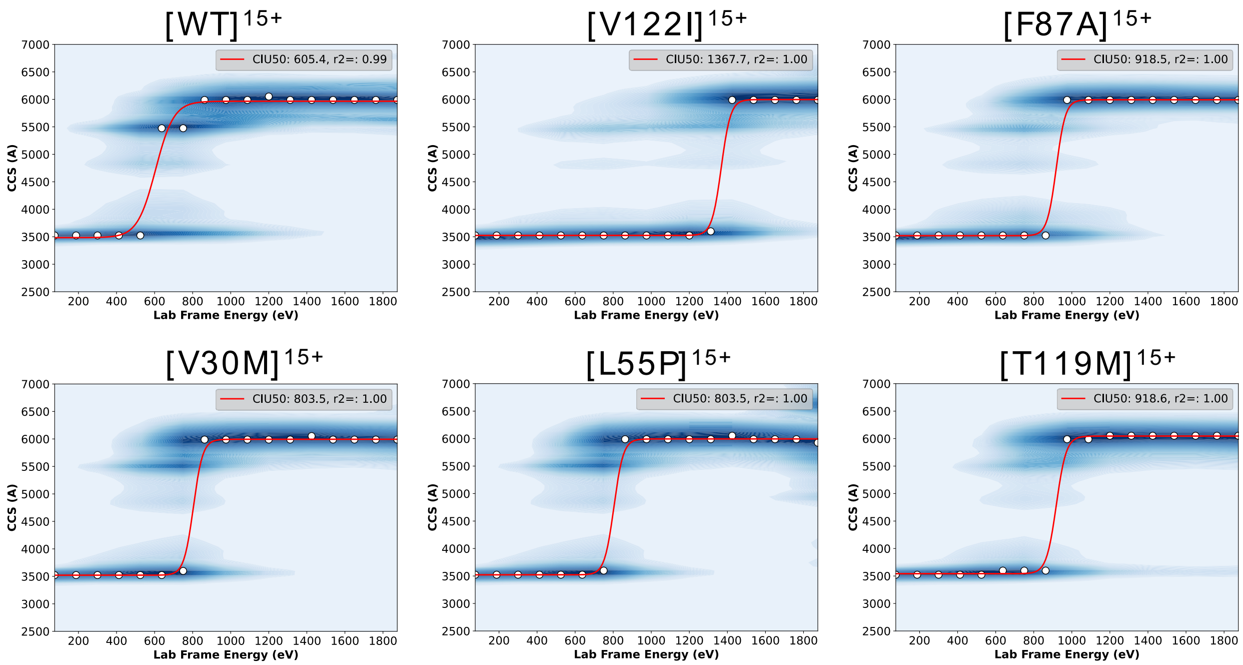

Figure S6. CIU50 plots of wt- and mutant TTR 15+ tetramer ions from CIU experiments using the standard fitting method in CIUSuite2. CIU50 was calculated from the smallest to the highest CCS, omitting any intermediate features.

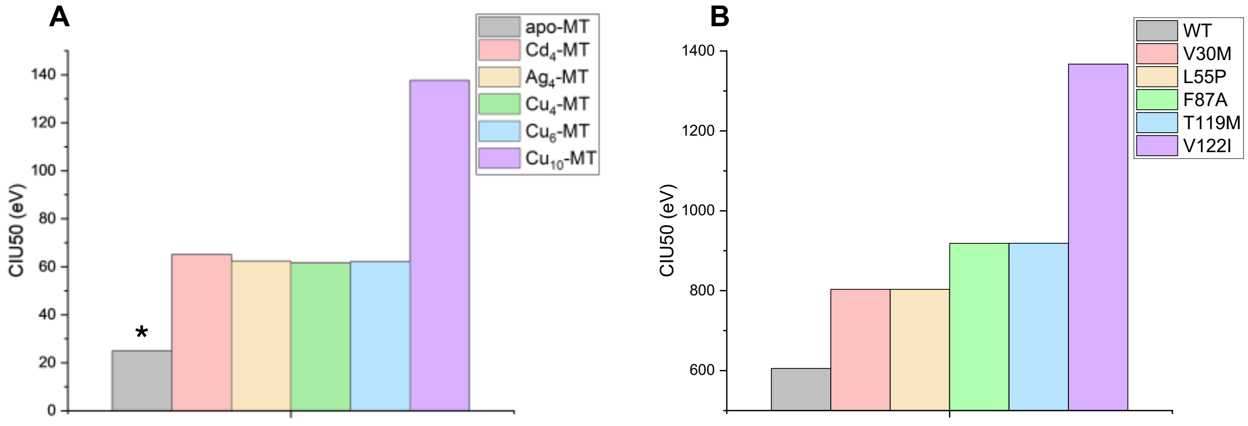

Figure S7. CIU50 values plotted from CIUSuite2 CIU50 plots for MT^{5+} **(A)** and TTR^{15+} **(B)** from Figures S5 and S6 respectively. (*) Apo-MT was primarily unfolded at the initial data point so we defined it’s CIU50 value as the lowest lab frame energy collected (25eV) as a reference only.

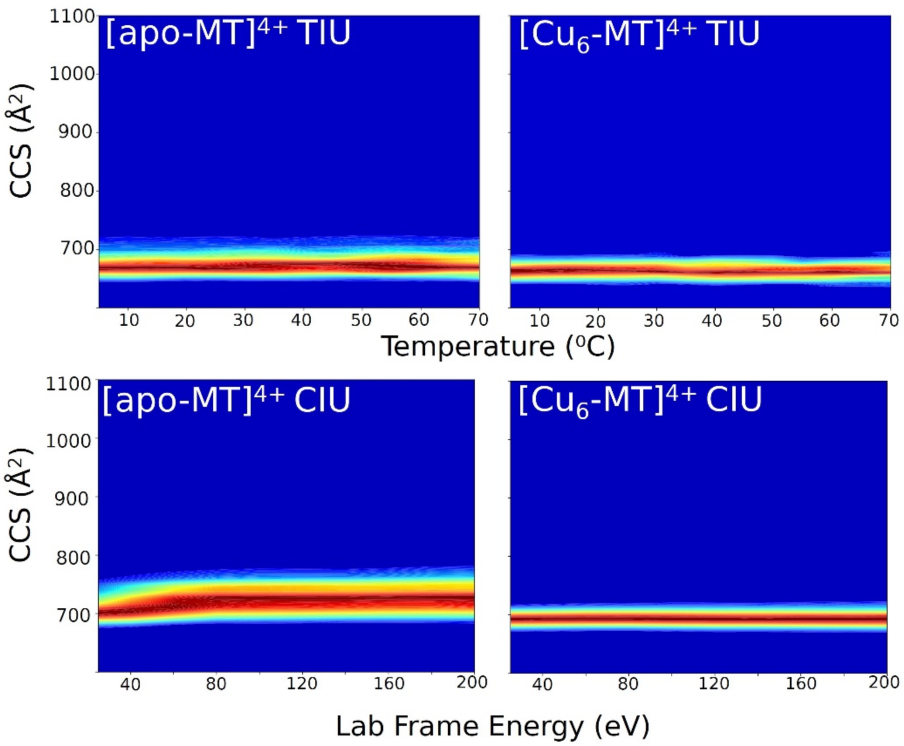

Figure S8. TIU and CIU heatmaps of apo-MT and Cu_6-MT 4+ charge state. Notice that there is no distinguishable change in CCS for either unfolding method which is not the case for the 5+ charge state CIU profiles seen in Figure S2. This trend is observed for all metalated MTs.

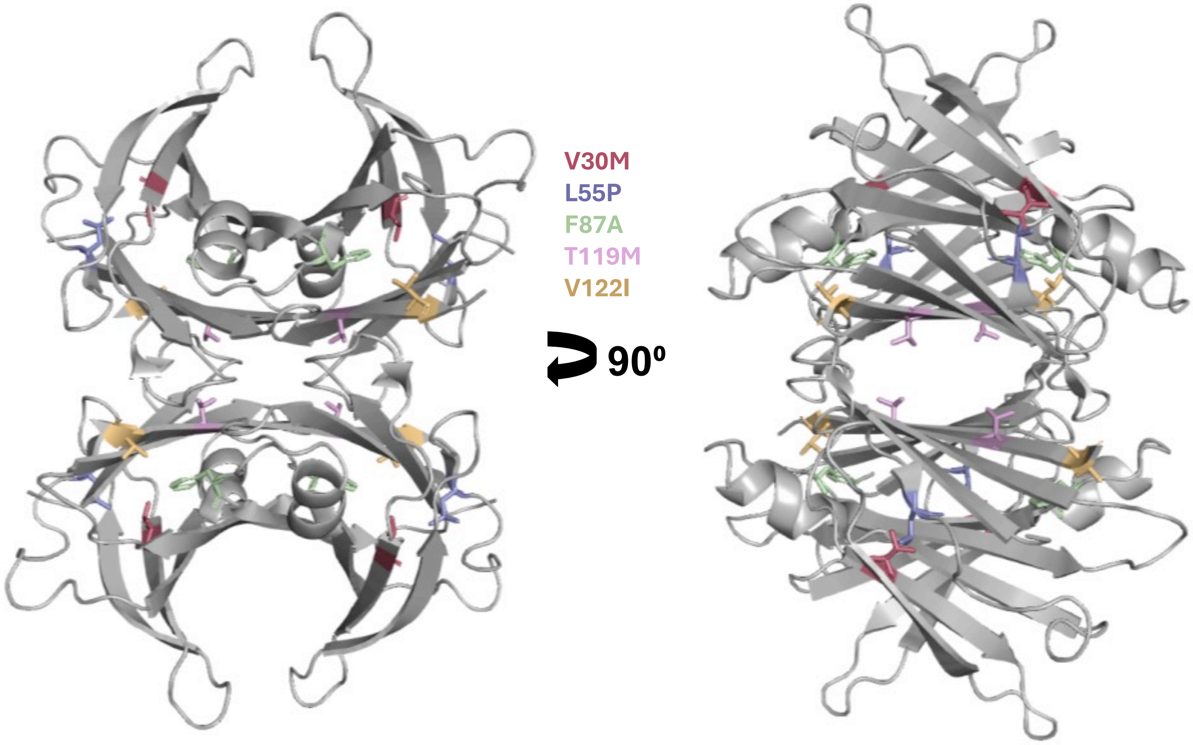

Figure S9. Two views of the x-ray crystallography structure of wt-TTR (PDB: 1DVQ) with each mutation site examined in this work colored and denoted in the figure. T119M is located within the dimer-dimer interface and thyroxine binding domain. F87A is located within monomer-monomer interface. V122I is involved in the monomer-monomer and dimer-dimer interface. Both V30M and L55P are located on the peripheral of the protein structure, away from any interfacial region.

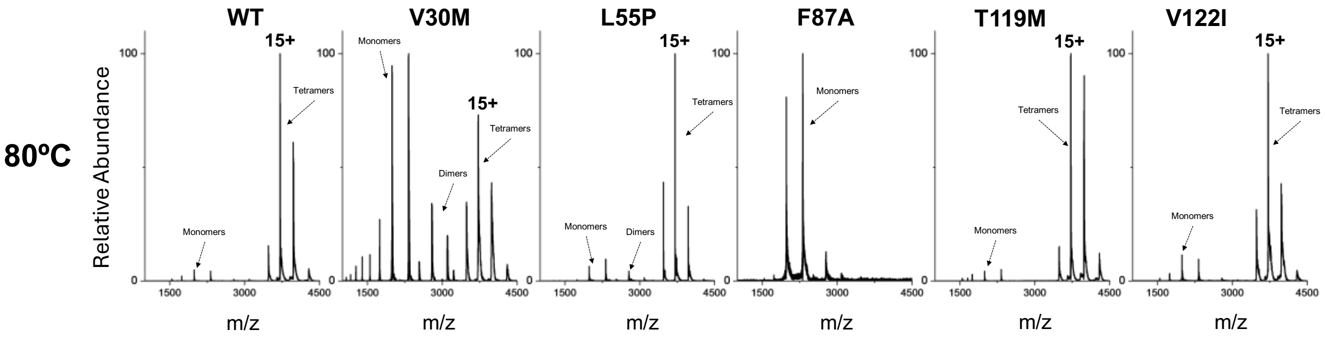

Figure S10. Representative mass spectra of wt and mutant TTR at 80 °C collected during vT-ESI experiments on the Thermo Fisher Q-Exactive UHMR. Tetramers, monomers, dimers and the highest tetramer charge state are labeled in the spectra. Note that F87A consists only of monomers after 65 °C due to its low tetramer stability relative to the other TTR mutants. Leading edges on the tetramers are truncations related to N-terminal thermo-cleavage studied previously.[1] Relative abundances of monomers and dimers were different across the 3 analytical replicates.

Table S1. Waters Synapt G2 Parameters for CIU Experiments

Synapt G2 Parameters	MT	TTR
Capillary (kV)	1.0 - 2.0	1.0 - 2.0
Source Temperature (ºC)	30-60	100
Sampling Cone (V)	10	15
Extraction Cone (V)	1	2
Trap Collision Energy (V)	5-50	5-125
Trap Gas Flow (mL/min)	0.40	4
Helium Cell Gas Flow (mL/min)	200	120
IMS Gas Flow (mL/min)	50-90	30
Trap Wave Velocity (m/s)	300	125
Trap Wave Height (V)	0.2-6	8
IMS Wave Velocity t (m/s)	300-310	400
IMS Wave Height (V)	6-20	18
Transfer Wave Velocity (m/s)	250	100
Transfer Wave Height (V)	5-15	1
Sample Collection Time (min)	2	2

Table S2. Waters Synapt G2 Parameters for TIU Experiments

Synapt G2 Parameters	MT	TTR
Capillary (kV)	1.0 - 2.0	1.0 - 2.0
Source Temperature (ºC)	30	30
Sampling Cone (V)	10	15
Extraction Cone (V)	1	2
Trap Collision Energy (V)	5	5
Trap Gas Flow (mL/min)	0.40	0.40
Helium Cell Gas Flow (mL/min)	200	100
IMS Gas Flow (mL/min)	70	30
Trap Wave Velocity (m/s)	310	125
Trap Wave Height (V)	6	8
IMS Wave Velocity (m/s)	250	400
IMS Wave Height (V)	5	18
Transfer Wave Velocity (m/s)	65	100
Transfer Wave Height (V)	2	1
Sample Collection Time (min)	2	2

Table S3. ThermoFisher Q-Exactive UHMR Parameters for Z_{avg} Measurement

UHMR Parameters	MT	TTR
Spray Voltage (kV)	1.0 - 2.5	1.0 - 2.5
Capillary Temperature	100	100
Source DC Offset (V)	21	21
In Source Trapping	Off	On
Desolvation Voltage (V)	0	-10
In Source CID (eV)	0	50
HCD CE	0	50
Trapping Gas Pressure Setting	3	4
Injection Flatapole DC (V)	5	8
Inter Flatapole Lens (V)	4	4
Bent Flatapole DC (V)	2	3
Transfer Multipole DC (V)	0	0
Detector m/z Target Optimization	Low m/z	Low m/z
Ion Transfer Target m/z	Low m/z	Low m/z
Sample Collection Time (min)	1	1

References:

(1) Shirzadeh, M.; Poltash, M. L.; Laganowsky, A.; Russell, D. H. Structural Analysis of the Effect of a Dual-FLAG Tag on Transthyretin. *Biochemistry* **2020**, *59* (9), 1013-1022. DOI: 10.1021/acs.biochem.0c00105.
